# Quantitative longitudinal imaging of activated microglia as a marker of inflammation in the pilocarpine rat model of epilepsy using [^11^C]-(*R*)-PK11195 PET and MRI

**DOI:** 10.1177/0271678X16653615

**Published:** 2016-01-01

**Authors:** J Yankam Njiwa, N Costes, C Bouillot, S Bouvard, S Fieux, G Becker, E Levigoureux, G Kocevar, C Stamile, JB Langlois, R Bolbos, C Bonnet, L Bezin, L Zimmer, A Hammers

**Affiliations:** 1 Neurodis Foundation, Lyon, France; 2 CERMEP-Imagerie du Vivant, Lyon, France; 3 Lyon Neuroscience Research Center, University Claude Bernard Lyon 1, Lyon, France; 4 Hospices Civils de Lyon, France; 5 CREATIS, Lyon, France; 6 Division of Imaging Sciences and Biomedical Engineering, King’s College London & Guy’s and St Thomas’ PET Centre, King’s College London, London, UK

**Keywords:** Epileptogenesis, TSPO, neuroinflammation, pilocarpine, status epilepticus

## Abstract

Inflammation may play a role in the development of epilepsy after brain insults. [^11^C]-(*R*)-PK11195 binds to TSPO, expressed by activated microglia. We quantified [^11^C]-(*R*)-PK11195 binding during epileptogenesis after pilocarpine-induced status epilepticus (SE), a model of temporal lobe epilepsy.

Nine male rats were studied thrice (D0-1, D0 + 6, D0 + 35, D0 = SE induction). In the same session, 7T T2-weighted images and DTI for mean diffusivity (MD) and fractional anisotropy (FA) maps were acquired, followed by dynamic PET/CT. On D0 + 35, femoral arterial blood was sampled for rat-specific metabolite-corrected arterial plasma input functions (AIFs). In multiple MR-derived ROIs, we assessed four kinetic models (two with AIFs; two using a reference region), standard uptake values (SUVs), and a model with a mean AIF.

All models showed large (up to two-fold) and significant TSPO binding increases in regions expected to be affected, and comparatively little change in the brainstem, at D0 + 6. Some individuals showed increases at D0 + 35. AIF models yielded more consistent increases at D0 + 6. FA values were decreased at D0 + 6 and had recovered by D0 + 35. MD was increased at D0 + 6 and more so at D0 + 35.

[^11^C]-(*R*)-PK11195 PET binding and MR biomarker changes could be detected with only nine rats, highlighting the potential of longitudinal imaging studies.

## Introduction

Immune and inflammatory mechanisms may play a fundamental role in the development of some forms of epilepsy.^1–4^ Several lines of evidence support this assumption like the activation of the immune system in some patients with seizure disorders or the high incidence of seizures in some forms of autoimmune encephalitis. It has also been reported that various injuries lead to microglial activation, including status epilepticus (SE) in rats.^5–7^

Non-invasive imaging of microglia activation biomarkers could be a relevant tool for detection, and especially for monitoring disease progression, therefore of great support to the evaluation of novel therapies^8^ especially for epileptogenesis.

The translocator protein 18 kDa (TSPO, previously the peripheral benzodiazepine receptor), is only lightly expressed in the healthy brain parenchyma while being drastically upregulated under neuroinflammatory conditions. This upregulation correlates with the activation of microglial cells, or the infiltration of blood-borne macrophages.^9^ Microglia are the resident immune cells of the central nervous system and are only activated in response to stimuli, thus acting as early sensors of brain pathology.^10^

*In vivo*, microglial activation can be detected using positron emission tomography (PET) ligands for the TSPO,^11–13^ and the reversible antagonist at TSPO [^11^C]-(*R*)-PK11195 is often used for studying diseases that involve microglial activation or the recruitment of macrophages as in multiple sclerosis,^14^ stroke,^15,16^ Alzheimer disease,^17^ traumatic brain injury,^18^ and to monitor brain lesions.^19^

SE, i.e*.* repetitive seizures lasting more than 30 min without regain of consciousness in between^20^ can cause an inflammatory brain response and a tendency for spontaneous seizures.^21^

Few studies have reported on TSPO imaging in epilepsy showing that [^11^C]-(*R*)-PK11195 binding was increased in Rasmussen’s encephalitis, but it was indistinguishable from controls in three patients with end-stage hippocampal sclerosis.^22^ A focally increased [^11^C]-(*R*)-PK11195 uptake corresponding to the seizure onset zone was identified in a patient with focal cortical dysplasia two years after an episode of focal SE.^23^ Using the TSPO ligand [^11^C]PBR28, higher standard uptake values (SUVs 60–120) were seen in temporal lobe structures ipsilateral to the seizure focus in 16 patients with unilateral temporal lobe epilepsy (TLE) at the group level, and 12/16 at the individual level.^24^ Preclinical studies have shown enhanced [^18^F]PBR111 binding in brain structures in the first week after kainate SE induction in rats.^6^ The potential use of TSPO PET as biomarker for the detection of drug refractoriness in TLE has also been reported recently in a rodent model.^5^

These results suggest potential clinical usefulness of TSPO tracers as sensor of brain status, as well as the possibility of studying the role of inflammation in the pathophysiology of epilepsy. For such studies, ideally, quantification in preclinical studies should be improved to the point that the study of each individual participant becomes possible, as is the case for many human studies now.

It has been claimed that quantification of [^11^C]-(*R*)-PK11195 binding is difficult because of its pharmacokinetic properties, poor bioavailability in brain tissue, and a relatively high level of non-specific binding.^25,26^ However, while quantitative analyses of TSPO ligand differences are scarce, one such analysis suggested substantially higher specific binding for [^3^H]PK11195 than [^3^H]PBR28 in tissue sectioned up to 14 days prior to use (see p. 1612 in Owen et al.^25^).

In this study, we investigated the ability of the PET ligand [^11^C]-(*R*)-PK11195 to detect microglia activation during epileptogenesis after pilocarpine-induced SE, a model of TLE,^27,28^ with rats as their own longitudinal controls. We addressed the challenging topic of quantitative analysis of the acquired dynamic [^11^C]-(*R*)-PK11195 PET images for a better understanding and interpretation of the acquired data.

## Materials and methods

### Overview

In this longitudinal study, nine Sprague Dawley rats (Harlan Laboratories, An Venray, The Netherlands; 244 ± 25 g at the beginning of the experiment) were studied with both magnetic resonance imaging (MRI) and PET/computed tomography (CT) at three different time points relative to induction of SE (D0): before SE (D0-1) and after SE (D0 + 6, D0 + 35). Imaging sessions were performed during the same short isoflurane anesthesia on the same bed. Young male adult rats were used to avoid hormonal changes and for optimal performance of the pilocarpine model. SE induction was performed early in the morning (8 a.m.).

Each animal was acting as its own control, with the aim of reducing the number of animals needed. One day after acquiring the initial baseline MRI and PET imaging, pilocarpine SE was induced. Subsequently, MRI and PET were acquired again at days 6 and 35 after SE induction. During the last PET scan (35 days after SE induction), arterial blood was sampled to obtain an individual arterial plasma input function (AIF) for data quantification of the last PET session, but also, after normalization, of the previous PET sessions. After the last PET sessions, rats were sacrificed.

All studies were carried out in accordance with European Communities Council Directive (2010/63/EU) as well as the recommendations of the French National Committee (2013/113). This study was reviewed and granted permission by the *Comité d’éthique pour l’expérimentation animale*, Neurosciences Lyon (CELYNE), reference number C2EA42-12-05-0501-001. The manuscript was written up in accordance with the Animal Research: Reporting in vivo Experiments (ARRIVE) guidelines (http://www.nc3rs.org.uk/arrive-guidelines; accessed 30 October 2015).

Animals were kept together in small groups (usually 2–3) in cages with a surface of∼2000 cm^2^ enriched with polyvinyl chloride tubing and wood to nibble. After SE, they were nursed back to strength and temporarily kept in individual cages, returning to group living as soon as possible. Individual rats were identified by their picric acid markings. Access to food and water was *ad libidum*. Animals were kept in a 12-h dark/light cycle.

### SE induction

Scopolamine methylnitrate (Sigma; 1 mg/kg) was given 30 min subcutaneously before intraperitoneal (i.p.) injection of pilocarpine (Sigma; 0.177 mg/cm^2^). Additional half doses were administrated subcutaneously to rats that did not reach SE after the first injection, an hour after the first dose, and a third dose half an hour after the second injection. Four of the nine rats needed only one dose of pilocarpine, three needed two doses, and one received three doses. Four rats reached SE, i.e. continuous seizures, at Racine stage 5^29^ (rats #1, 2, 6, 9) four at stage 4 (rats #3, 4, 5, 7), and one at stage 3 (rat #8).

Two hours after the onset of SE, diazepam (10 mg/kg i.p.; Roche) was given, followed by another dose of 5 mg/kg, given 1 h later subcutaneously.

There was no mortality due to SE.

### Animal preparation for imaging sessions

Rats were anesthetized with isoflurane (4% induction, 1.75–2.25% maintenance), and a venous catheter was inserted into the tail vein for PET tracer administration. The customized scanning MRI and PET compatible plastic support bed (Bruker Biospec Animal Handling Systems) was equipped with a warm water recirculation system. The temperature was maintained at 37℃, and the breathing rate was monitored throughout the experiment. Prior to the third PET scan, a femoral artery was catheterized under isoflurane anesthesia, with additional local analgesia with lidocaine (5 mg/kg).

### MR imaging

Magnetic resonance (MR) experiments were carried out using a Bruker BioSpec 7 T (Bruker BioSpin MRI, Ettlingen, Germany), a small-animal MR system operating at 300.26 MHz. T_2_ rapid acquisition with relaxation enhancement (RARE) sequences were used for acquiring structural images of the rat brain. The parameters used for the acquisition of coronal brain slices were the following: TE/TR = 69.1/13,445.8 ms, FOV = 3 × 1.5 cm^2^, matrix dimension 256 × 128, slice thickness 0.4 mm, number of slices = 75, RARE factor = 8.

Subsequently, echo planar imaging (EPI)-based diffusion tensor images (DTI) were acquired to assess microstructural mechanisms underlying neuroinflammation after the brain insult. The acquisition parameters were as follows: TE/TR = 19.3/6250 ms, diffusion directions = 30, number of A0 images = 5, FOV = 3 × 1.5 cm^2^, matrix dimension = 128 × 64, slice thickness = 0.8 mm, number of slices = 25.

The mean diffusivity coefficient (MD) and the fractional anisotropy (FA) maps were derived using the DTIfit toolbox, a module of FMRIB's Software Library.^30^

### PET imaging

Following the MRI scans, animals were kept under anesthesia (except for the last scan, where the PET examination was performed a few hours after MRI, with the rat in supine rather than prone position as for the MRI in order to facilitate the arterial blood sampling procedure) and transported on the same bed to the micro PET/CT scanner room (Siemens Inveon). For each rat, a CT scan was acquired for attenuation correction and registration. A mean activity of ∼ 37 MBq of [^11^C]-(*R*)-PK11195 was injected as a bolus via the venous catheter, simultaneously with the start of the dynamic PET acquisition.

Data were acquired in list mode over 60 min after injection and rebinned in frames of 9 × 20; 4 × 60; 4 × 120; and 3 × 900 s. Images were reconstructed by filtered back-projection with a voxel size of 0.38 × 0.38 × 0.79 mm^3^.

### Blood sampling

Femoral arterial blood was sampled at D0 + 35 to derive rat-specific metabolite-corrected plasma input functions for kinetic modeling. Thirteen blood samples (200 µl) were collected through the femoral artery catheter, initially at 3, 10, 20, 30, 40, 50 s, and then at 1, 2, 5, 10, 25, 40, 50 min post-injection.

Four samples (5, 25, 40, and 50 min) were used to analyse metabolites. After centrifugation of the blood samples during 5 min at 5000 *g*, plasma was extracted and analysed with solid-phase extraction followed by HPLC chromatography with online radioactivity and UV detectors set at 310 nm (Merck Hitachi). Fifty micro litre (µl) plasma samples were mixed with 150 µl of acetonitrile and 50 µl of unlabelled [^11^C]-(*R*)-PK11195. After centrifugation, the supernatant was withdrawn and filtered using 0.45 µm filters. Twenty micro litre of this filtered composition was passed through HPLC at 1 ml/min with a mobile phase of H_3_PO_4_ 0.1 M/CH_3_CN (7:3). Twenty HPLC elution fractions were then collected and radioactivity quantified with a Cobra gamma counter. The parent fraction curve was fitted with a biexponential function. with *f_p_* standing for the tracer not bound to plasma proteins. The fraction computation assumes negligible metabolites before a *Begin* time point (parent concentration = 1).

**Figure:**
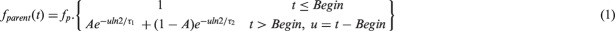

The AIF of the unmetabolized tracer in plasma was generated by multiplying the total plasma input function with the result of the parent ratio fitted function.^31^ This AIF was used for compartmental modeling of the D0 + 35 PET time activity curves (TACs). The AIF were derived for each of the seven rats which underwent arterial blood sampling. For the baseline, and for the D0 + 6 scan, a derived AIF was computed by normalization of the D0 + 35 AIF. The normalization was obtained as follows

(2) $${\mathrm{AIF}}_{D0+i}={\mathrm{AIF}}_{D0+35}\frac{{\mathrm{SUV}}_{D0+i}}{{\mathrm{SUV}}_{D0+35}}$$

with SUV standing for SUV of the activity at each time points

(3) $$\mathrm{SUV}=\frac{\mathrm{Concentration}(\mathrm{Bq}/\mathrm{ml})}{\mathrm{Injected}\mathrm{dose}(\mathrm{Bq})}\times \mathrm{weight}(g)$$

For the computation of the mean AIF at each time point, equation (2) was used replacing AIFD0 + 35 by the normalized mean AIF. Normalization was obtained by initially rescaling between min and max of all the AIFs at D0 + 35. They were then rescaled using the mean weight and injected dose corresponding to the mean AIF.

The arterial whole blood activity curve of D0 + 35 was obtained as follows. From the 13 discrete arterial samples was derived the plasma over blood ratio modeled by a straight line as in Jenkinson et al.^31^ Whole blood activity curves of baseline and D0 + 6 scans were obtained with the normalization procedure using equation (2). The arterial whole blood activity for each sample was multiplied with the ratio to obtain the total plasma activity concentration at each time point.

### Image processing

Within and between subject comparisons were performed at a brain regional level. Brain region parcellation was obtained using an automated multi-atlas image segmentation approach accounting for normal and atrophic rat brains. The multi-atlas propagation with enhanced registration (MAPER) algorithm,^32,33^ which has been developed to accurately segment normal and atrophic human brains, was adapted for this aim. MRIs of a multiple rat dataset of 7 MRIs and atlases containing 29 regions each (independent of the present dataset but acquired on the same MR scanner^34^) were registered to the MRI space of each individual rat time point based on MAPER principles, and then registered to the individual CT for the region-of-interest propagation and fusion of labelled images of the atlas dataset onto individual PET images.

The non-rigid NiftyReg registration algorithm of the NiftyReg software^35^ was used for MRI to MRI registrations, and SPM8 (Wellcome Trust Centre for Neuroimaging, www.fil.ion.ucl.ac.uk/spm) was used for registration between MRI and CT images.

We hypothesized that inflammation would be most pronounced in three temporal regions (the hippocampus, the amygdala, the temporal cortex) and the thalamus.

### PET kinetic modeling

*In vivo* quantification of tracer kinetics was performed using the dynamic PET images representing the local concentration of the exogenous [^11^C]-(*R*)-PK11195 ligand in the tissue. Several kinetic models were assessed for [^11^C]-(*R*)-PK11195 regional TAC modeling.

Two groups of models were used. The first group consists of methods using arterial blood samples and plasma metabolite-corrected AIFs. They provide quantitative total volume of distribution (V_T_) of tracer in tissue. Among them, the Logan plot (LP)^36^ method is based on a graphical solution of the partial derivative equations of the compartment model of tracer distribution in tissues. The second method (one tissue class – 1TC – model) describes the bidirectional flux of tracer between blood and tissue. It uses non-linear fitting of PET TACs on algebraic solutions of the partial derivative equations of the compartmental model. We also evaluated the two-tissue class model, but this led to a very large number of model failures in the regions examined, and it was not further pursued (data not shown).

The second group of methods does not need an AIF, but uses a reference region (assumed to be devoid of specific binding). The Logan reference tissue (LR) method^37^ and the simplified reference tissue model (SRTM)^38^ were assessed. These methods provided the non-displaceable binding potential (BP_ND_) of the ligand in the tissue. Reference tissue methods were validated against methods using AIF. The variability of the kinetic parameters and the magnitude of the percentage increase after SE were taken into account to assess the efficiency and the robustness of the models.

Quantification of kinetics was performed using the Pmod (http://www.pmod.com/technologies/index.html) tools for image processing and general kinetic modeling.

Correlation coefficients ${\overset{\wedge }{\text{r}}}_{\text{p}}$, ratio of the covariance between two variables and the variance of each variable, were computed for some assessments.

## Results

**Figure 1.**
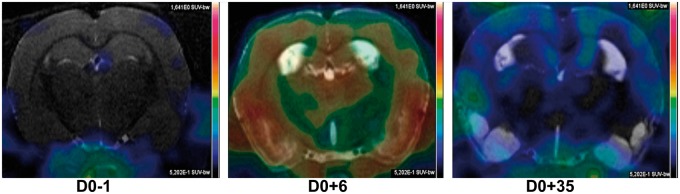
The effects of inflammation on brain structures. [^11^C]-(*R*)-PK11195 PET total sum images are superimposed on coronal MRI selected slices for a representative rat. D0-1 is baseline image; D0 + 6/D0 + 35 images were acquired 6 and 35 days after SE induction. Images are scaled to the same colour scale.

The imaging time points (baseline, D0 + 6 and D0 + 35 after SE induction, i.e. healthy, acute and chronic stages of TSPO expression) could be visually differentiated by the brain structures’ ligand binding on summed radioactivity images (see Figure 1), dynamic images, and TACs. There was almost no binding at baseline as expected confirming the low expression of TSPO in the healthy brain. After SE, an upregulation of microglia was observed during epileptogenesis (six days after SE induction) and lower at the chronic phase of epilepsy, here 35 days after SE.

### Model comparisons

The brainstem had the least variation between rats and time points and was used as reference region for the kinetic modelling with the LR and SRTM models. We have also run the SRTM with cerebellum as the reference region, with overall higher variability at all time points (data not shown). Distribution volume ratios (DVRs corresponding to BP_ND_ + 1) were computed for the assessment of the models in order to avoid negative numbers. Especially at baseline, BP_ND_ may assume negative values (i.e. the binding potential of the target region is lower than that of the reference region) due to noise and the near-absence of TSPO expression in the healthy brain.

Quantification of [^11^C]-(*R*)-PK11195 binding in the brain was possible in all brain datasets and all 29 regions per brain with three of the four models (see below for details). The intersubject variability was below 20% in all cases, compatible with expected normal biological variation, except for the SRTM model at baseline (35%). Results are summarized in Table 1.

**Table 1.** Comparison of kinetic parameters derived from the Logan plot (LP), the 1 tissue compartment (1TC) model, the simplified reference tissue model (SRTM) and the Logan reference (LR) model.

	LP (V_T_)	1TC (V_T_)	SRTM (DVR)	LR (DVR)
D0-1	3.2 ± 0.5	2.6 ± 0.4	0.8 ± 0.3	0.9 ± 0.1
D0 + 6	5.6 ± 0.9	4.8 ± 0.9	1.2 ± 0.2	1.3 ± 0.2
	**+75%**	**+86%**	**+49%**	**+39%**
D0 + 35	3.3 ± 0.7	2.6 ± 0.4	1.1 ± 0.1	1.1 ± 0.1
	**+4%**	**+2%**	**+28%**	**+17%**

Note: An arterial input function is used for the LP and the 1TC. The reference region used for the SRTM and LR is the brainstem.

Values are the mean value of the mean values of each rat in 26 ROIs (excluding the ventricles) at each time point ± mean values of standard deviation for each rat in 26 ROIs at each time point.

Note that percentage increases in binding given in bold are in comparison to the baseline.

DVR: Distribution volume ratio.

In terms of inter-regional variability, in almost all time points and for every rat (data not shown), the SRTM model yielded a high coefficient of variation (>20%). This variability was lower with the other models in almost all animals at every time point (<20%) except in two rats (rat #1 at D0 + 6, rat #6 at D0 + 35) for the LP/1TC/LR models and in an additional rat for the LR model (rat #6 at baseline). The SRTM model failed in some structures, five times at baseline and once at D0 + 6 and D0 + 35 after SE induction. There was no failure with the other three models. Thus, the results derived with the LP, 1TC and LR were more robust than those derived with the SRTM.

**Figure 2.**
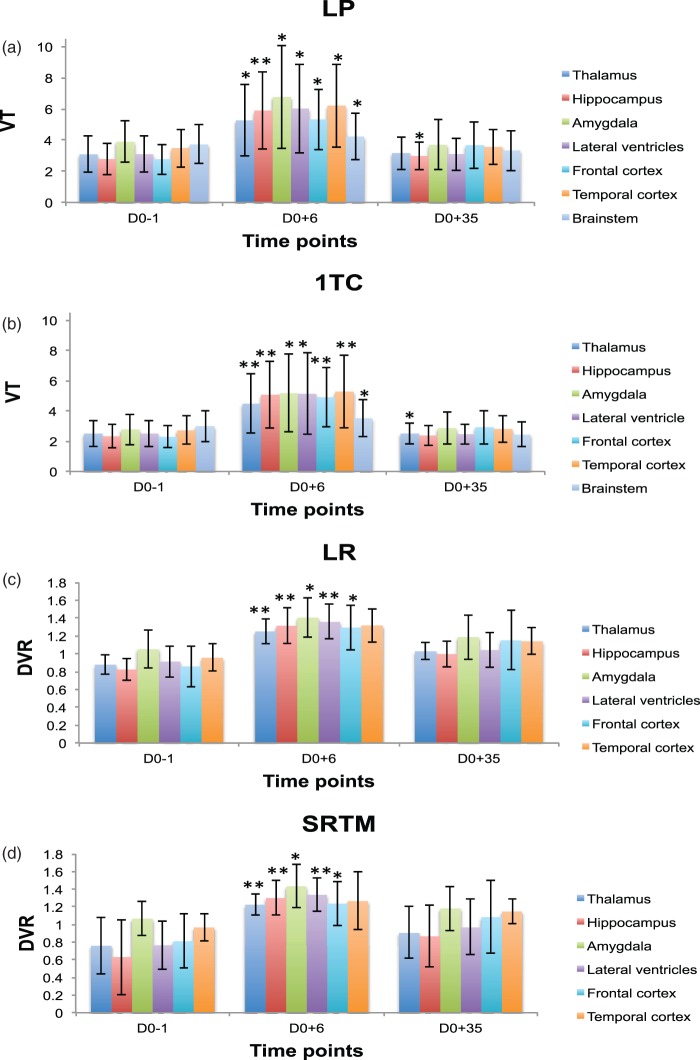
Mean left/right regional V_T_ and DVR (mean ± SD) time course data before (D0-1) and after (D0 + 6, D0 + 35) pilocarpine-induced SE in selected brain structures (* highlights p values < 0.05 and ** for p < 0.009 computed with t test against baseline): (a) Logan plot model, (b) one tissue compartment model, (c) Logan reference model, (d) simplified reference tissue model. Changes were considered significant in a structure when they were significant both on the left and the right side of the given structure. Seven rats had arterial input functions and could be analysed with the 1TC and LR models; all nine could be used for the LR and SRTM models.

Quantification of [^11^C]-(*R*)-PK11195 binding to activated microglia showed similar significant increases of the kinetic parameters computed at D0 + 6 (p < 0.009) for all regions expected to be affected (three temporal regions: hippocampus, amygdala, temporal cortex, and thalamus) with all quantification methods used in this study. From D0 + 6, parameters returned towards baseline values after 35 days. This decrease was significant (p < 0.05) from day 6 to day 35 after SE with all four models used. While the kinetic parameter variations were more marked in the regions expected to be most affected in the epilepsy model, i.e. temporal/limbic regions, changes also occurred in the frontal cortex and, to a lesser degree, in the brainstem, indicating widespread changes. The results are illustrated in Figure 2.

In the brainstem, changes were minor, just significant using a parametric test (Student t test), and not significant using a non-parametric test (Mann-Whitney U). This consolidated our choice of this region as a reference region (Table 2).

**Table 2.** 1TC modeling: mean (left/right) regional V_T_ (mean ± std) values before (D0-1) and after (D0 + 6, D0 + 35) pilocarpine induced SE in seven rats.

	Thalamus	Hippocampus	Amygdala	Temporal cortex	Frontal cortex	Brainstem
D0-1	2.5 ± 0.9	2.3 ± 0.8	2.8 ± 1.0	2.7 ± 0.9	2.3 ± 0.7	3.0 ± 1.0
D0 + 6	4.5 ± 2.0	5.1 ± 2.2	5.2 ± 2.6	5.3 ± 2.4	4.9 ± 2.0	3.5 ± 1.2
	(p = 0.03)**	(p = 0.02)**	(p = 0.13)*	(p = 0.02)**	(p = 0.007)**	(p = 0.3)
	**79%**	**118%**	**86%**	**94%**	**114%**	**17%**
D0 + 35	2.5 ± 0.7	2.4 ± 2.2	2.9 ± 1.1	2.8 ± 0.9	2.9 ± 1.1	2.4 ± 0.8
	(p = 0.94)	(p = 0.83)	(p = 0.83)	(p = 0.62)	(p = 0.23)	(p = 0.13)
	**−44%**	**− 53%**	**− 44%**	**− 47%**	**− 40%**	**− 31%**

Note: Expressions in brackets are the highest p values derived from U tests for either left or right side of the structure. Asterisks (*left or right side, ** both sides) are highlighting structures where the p values indicated significant (p < 0.05) changes. Values are based on the seven rats which had arterial input functions. Beside the regions hypothesised to show changes, frontal cortex and brainstem are shown as regions expected to show less change. Percentage increases of V_T_ at D0 + 6 compared to the baseline and percentage decreases of V_T_ from D0 + 6 to D0 + 35 are given in bold in the corresponding cells.

Note that the percentage changes are expressed relative to the earlier time point.

The overall results obtained from the four kinetic models used in this study suggest that the availability of blood input samples helps estimating more consistent kinetic parameters.

### Biological results

Given the model comparisons in the preceding sections, in the following, we will only discuss results obtained with the AIF-based model 1TC, which had lower V_T_ dispersion in the different brain structures than the LR model.

The 1TC model detected large V_T_ increases on day 6 after SE: 118% in the hippocampus, 86% in the amygdala, 94% in the temporal cortex, and 79% in the thalamus, but only 17% in the brainstem. Significant decreases of V_T_ (p < 0.05, U test) were also observed from day 6 to day 35 after SE induction in all structures except in the brainstem. The V_T_ decreases relative to the peak at day 6 were 53% in the hippocampus, 44% in the amygdala, 47% in the temporal cortex, 44% in the thalamus and 31% in the brainstem (Table 2). Only the thalamic V_T_ remained significantly higher than at baseline at day 35 (Figure 2).

It was also possible to demonstrate that structural changes occurred in the lateral ventricles, visible on MRI acquisitions. The lateral ventricles enlarged by 43% at day 6 and by 91% at day 35 after SE induction in comparison to the baseline. Changes in lateral ventricle volumes were highly significant (p < 0.005) at D0 + 35 and already significant (p < 0.05) at D0 + 6 compared to the baseline. The continuing enlargement of the ventricles did not show a high correlation with the increases of V_T_ at D0 + 6 and D0 + 35 regarding the baseline. The correlation coefficient in terms of the percentage changes in V_T_ and the percentage enlargement of the ventricles from the baseline to D0 + 6 was ${\overset{\wedge }{\text{r}}}_{\text{p}}=0.40$ and ${\overset{\wedge }{\text{r}}}_{\text{p}}=0.36$ between the baseline and D0 + 35.

**Figure 3.**
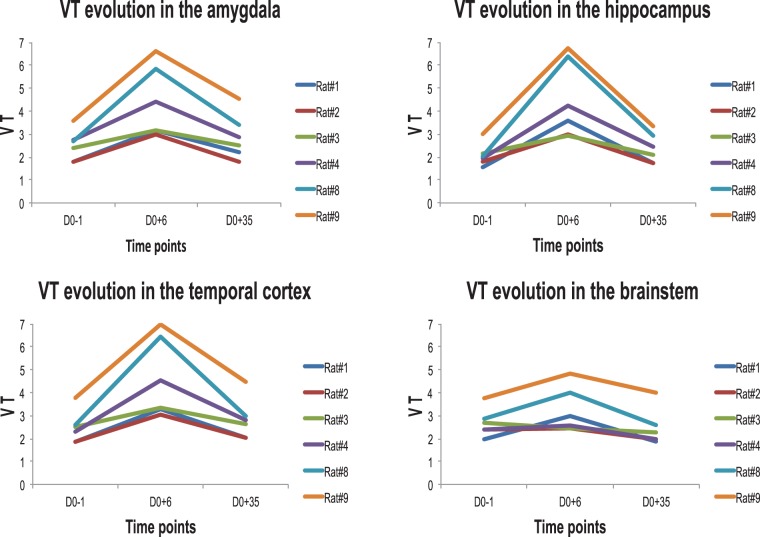
The trend of VT changes, in the 1TC model, for individual rats at each time point of the longitudinal study, i.e. baseline (D0-1), D0 + 6 and D0 + 35 after SE induction. The results of rat #5 which had an AIF available is not shown because information at D0 + 35 were not extracted for this rat due to the corresponding MRI dataset being unusable.

The evolution of the V_T_ values for the 1TC model had the same trend in the temporal/limbic regions, expected to be affected by SE, in all rats. In contrast, not all rats did show such a trend in the brainstem (Figure 3).

**Figure 4.**
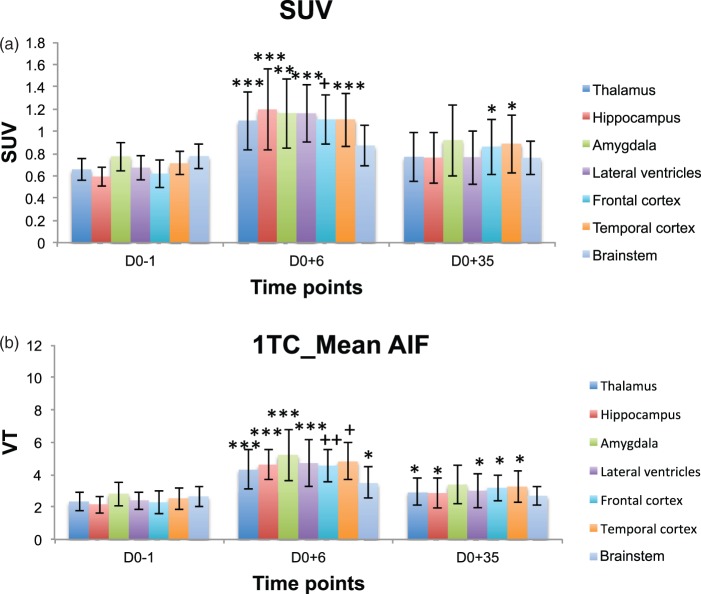
Mean left/right regional VT and SUV (mean ± std) time course data before (D0-1) and after (D0 + 6, D0 + 35) pilocarpine-induced SE in selected brain structures (* highlights p values < 0.05 and ** for p < 0.005, *** for p < 5*10 ^− 4^, + for p < 5*10^−5^, ++ for p < 5*10^−6^ computed with t test against baseline): (a) SUV, (b) 1TC – one tissue compartment model using a normalised mean arterial input function (normalised according to the injected dose and the weight of each rat, and averaged over seven rats). Changes were considered significant in a structure when they were significant both on the left and the right side of the given structure.

Using the average AIF, changes in V_T_ showed similar trends as when using individual AIFs (see Figures 4(b) and 2(b)). Highly significant increases (p < 0.0005) in V_T_ were observed as following six days after SE induction: 115% in the hippocampus, 86% in the amygdala, 92% in the temporal cortex, 85% in the thalamus. The changes in the brainstem were 32% (p < 0.05). Decreases in V_T_ values were detected in comparison to the top reached at day 6 after SE induction of 39% in the hippocampus, 35% in the amygdala, 32% in the temporal cortex, 32% in the thalamus, and 23% in the brainstem. These decreases were significant (p < 0.0005). However, and in contrast to the results obtained with individual AIFs, V_T_s at D0 + 35 remained significantly higher than at baseline in several structures.

SUVs showed similar trends in variation observed regarding each considered time point (baseline, D0 + 6 and D0 + 35 after SE induction), see Figure 4(a). Significant increases were detected from the baseline at day 6 as following in the different brain structures: 101% in the hippocampus, 50% in the amygdala, 55% in the temporal cortex, 66% in the thalamus and only 23% in the brainstem. Decreases from D0 + 6 to D0 + 35 were 72% in the hippocampus, 62% in the amygdala, 55% in the temporal cortex, 74% in the thalamus, and 117% in the brainstem. However, and again in contrast to the results obtained with the 1TC model with individual AIFs, SUVs at D0 + 35 remained significantly higher than at baseline in two structures.

While the magnitude of the changes in V_T_ obtained with the 1TC model and either individual or mean AIFs and changes in SUV were broadly similar at D0 + 6, the intersubject variability was much reduced when not using the individual AIF. In addition, the “gold standard” 1TC model with individual AIF did not show increases relative to baseline, whereas both the 1TC model with mean AIF and SUVs did (Table 3).

**Table 3.** Comparison of two methods not requiring individual arterial input functions against the reference method 1TC.

Brain structures	D0 + 6	D0 + 35
1TC		
Hippocampus	118% (0.44)	2% (0.28)
Amygdala	86% (0.49)	3% (0.37)
Temporal cortex	94% (0.45)	4% (0.32)
Thalamus	79% (0.44)	0% (0.28)
Brainstem	17% (0.35)	−19% (0.33)
1TC_mean AIF		
Hippocampus	85% (0.28)	25% (0.28)
Amygdala	86% (0.31)	21% (0.31)
Temporal cortex	92% (0.23)	30% (0.30)
Thalamus	85% (0.28)	25% (0.28)
Brainstem	32% (0.27)	2% (0.22)
SUV		
Hippocampus	101% (0.30)	28% (0.30)
Amygdala	50% (0.27)	19% (0.35)
Temporal cortex	55% (0.21)	25% (0.20)
Thalamus	66% (0.26)	17% (0.22)
Brainstem	12% (0.19)	−2% (0.15)

Note: Percentage changes relative to baseline. Coefficients of variation (standard deviation/mean) are given in brackets.

ITC: 1 tissue compartment.

In terms of DTI-derived diffusion coefficients, there was a decrease in FA values at D0 + 6 and a recovery at D0 + 35. The MD increased at D0 + 6 and continued to do so at D0 + 35.

The correlation coefficient between the percentage of changes in V_T_ and in FA was ${\overset{\wedge }{\text{r}}}_{\text{p}}=0.68$ at D0 + 6 and ${\overset{\wedge }{\text{r}}}_{\text{p}}=0.74$ at D0 + 35. This coefficient was smaller regarding the MD values (${\overset{\wedge }{\text{r}}}_{\text{p}}=0.07$ at D0 + 6 and ${\overset{\wedge }{\text{r}}}_{\text{p}}=0.3$ at D0 + 35).

## Discussion

The ability to detect [^11^C]-(*R*)-PK11195 PET binding changes after SE was validated in this longitudinal study of microglia activation in a rodent model of TLE. This detection was possible with the seven rats which had an AIF derived from blood sampling at D0 + 35 and with all nine rats in which using a tissue reference region was possible.

This study showed, based on qualitative and quantitative assessments, that differentiation of baseline PET images from images of the time points following SE induction was possible. The AIF, corrected for metabolites, allows an estimation of tracer delivery to the brain, and is the gold standard for absolute quantification. Therefore, the quantification results were strengthened by the availability of individually derived AIFs. The choice of the 1TC with individual AIFs as the method of reference was also motivated by the finding of relatively low inter-rat variability at baseline as expected, high interindividual variability after status (as expected after any insult), and as a result the best differentiation between these two states (Figure 2).

The longitudinal design of the study has permitted the detection of interindividual differences. For example, rats which had a slightly higher baseline V_T_ (rats #4, #8, and #9 in Figure 3) had a stronger inflammatory reaction in the expected areas than the other rats. This might be related to interindividual differences in the severity of epilepsy in the chronic phase. While these could not be quantified in the present work, they would be an interesting area for future study.

We could directly check involvement of the reference region chosen, i.e. the brainstem, via the independent AIF. While changes were much smaller than in the regions expected to show changes, they were present in at least some rats (Figure 3). Our finding of better performance of the brainstem compared with the cerebellum will not necessarily hold in other models, and scientists should check their assumptions in those situations. When using a reference region in the absence of pathology, BP_ND_ may be negative in the absence of pathology for at least half of regions (Figure 2 (c) and (d)). While the cerebellum is often used as reference for various tracers, in this study, it provided results with higher dispersion than the brainstem (data not shown, see comment in Results section). The use of DVR helped mathematically with the calculation of measures of spread, notably the standard deviation.

Although previous epilepsy studies in humans^22–24^ and animals^5^ have shown increased [^11^C]-(*R*)-PK11195 uptake in temporal regions and those affected by inflammation, none of them have performed kinetic modeling for the quantification of the acquired data. It has been also shown in this study that despite the partial volume effects probably affecting neighboring voxels, [^11^C]-(*R*)-PK11195 had enough specific signal to distinguish between healthy and inflammatory stages.

Increased V_T_ values reflect microglia activation related to brain inflammation, early after SE induction, corresponding to epileptogenesis. The increase of V_T_ is generally interpreted as an increase in activated microglia but could also be due to reactive astrocytes and peripheral macrophages crossing the blood brain barrier after SE and persisting in the brain days after SE.^3^ While a prior study in humans^24^ found that medial temporal TSPO expression was due primarily to choroid plexus, binding increases in our study were generally far away from the ventricles and hence the choroid plexus (Figure 1).

The decreases of V_T_ towards baseline observed in the chronic epilepsy phase (D0 + 35) may correspond to a return to normal regulation of microglia in the brain. In a different model of TLE (kainic acid-induced SE), the peak of microglial activation as assessed with [^3^H]PK11195 autoradiography was at two weeks, with a slow return towards baseline values but with some persistent activation in the chronic phase.^20^ It is therefore possible that the more widespread activation seen with the 1TC model with average AIF corresponds more closely to biological reality, even if the use of the average input function seems to blunt interindividual differences in response (cf. Figures 2(b) versus 4(b)). Such interindividual differences are to be expected as in any pathology and are required to study the relationship between imaging and epilepsy parameters.^39^ A limitation of our study is that due to infrastructure limitations we were unable to quantify seizures in this study. Notably, we had not implanted electroencephalography electrodes as they lead to local inflammation with microglial activation (L Bezin, personal communication).

Simple weighted images of tissue radioactivity (SUV) did show the overall time course (Figure 1), significant increases at D0 + 6, and an – albeit slight – expected increase in variability at D0 + 6. While SUVs will be vulnerable to changes in peripheral clearance of [^11^C]-(*R*)-PK11195, for example in a model with a pharmacological intervention, they remain the most easily implemented option for simpler models, requiring only careful calibration of the scanner and precise determination of animal weight and (cross-calibrated) injected dose.

PET results were consolidated by MRI biomarkers: in the acute phase, V_T_ increases reflecting microglia activation and related to brain inflammation corroborate structural damage related to the SE insult resulting in decreased FA and increased MD. V_T_ and FA recovery may reflect remaining axonal structures and decreasing oedema in brain structures. The continuing increases of the MD in the chronic phase of epilepsy could be the result of long-term activation of microglia combined with cell death. This continuing increase of the MD values at D0 + 35 may reflect presence of microglial scar related to neurodegenerative processes and unsuccessful axonal regeneration which is a common phenomenon following acute injury of the central nervous system.^40^ This phenomenon may also explain why the percentage increases in ventricle size do not correlate with the percentage increases in V_T_.

The significant changes of the axial diffusivity observed at D0 + 35 in all the structures expected to be affected except the amygdala, but including the white matter, may be explained by axonal degeneration in the concerned structures. The important changes in the values of radial diffusivity observed at D0 + 6 and D0 + 35 describe persistent structural damage and may reflect hippocampal sclerosis.

Almost all of the brain regions showed increased [^11^C]-(*R*)-PK11195 binding and changes in DTI parameters. On our point of view, this is suggested to be due to the diffuse distribution of the pilocarpine in the brain in contrast to electrical stimulation, which has a more focal effect.

Quantification of PET data acquired in this study performed through modelling with an average AIF showed results in line with those obtained with individual AIF. These results open perspectives to use this mean AIF in other studies and avoid the invasive nature of the arterial blood sampling and sacrifying the animals after the procedure. However, the good apparent performance of the average AIF method may be due in part to an artificial decrease in intersubject variability through the use of the same (albeit scaled) input function for all animals (cf. Figures 2(b) versus 4(b) and discussion above).

The conventional arterial blood sampling is invasive, noisy due to the small volumes of blood available, and technically difficult to set up. Further, as our quantification results have shown, it is not always obvious to determine a reference region free from tracer binding. Notwithstanding, SUV (Figure 4), LR, and SRTM (Figure 2) were able to show significant and systematic differences, and may be sufficient for answering many biological questions.

The SRTM model could in principle be extended to supervised reference tissue extraction^30^ with the available data. Our pilot studies exploring this option had been encouraging,^41^ but there were also serious limitations, notably due to the order of magnitude fewer voxels per tissue volume unit available in rat PET data compared to human PET, and the resulting much more severe partial volume effects (unpublished data). While we did not exhaust this or indeed all SRTM options, using cerebellum rather than brainstem led to much more variable BP_ND_ estimates even at baseline (data not shown). A two-compartment model has also been used recently^42^ for quantifying changes in V_T_ values in human patients with TLE, compared to healthy controls, using [^11^C]PBR28 PET. However, in our study, the two-compartment model did not perform well.

Other future work could include the assessment of image derived AIFs via multimodal imaging^43^ and comparisons with the results obtained here.

[^11^C]-(*R*)-PK11195 PET was able to detect TSPO upregulation reflecting neuroinflammation, following pilocarpine-induced SE. Quantitative binding changes could be detected with only 7–9 rats, complementing MRI biomarker results.
